# Infantile Paralysis—A Plea for the Better Understanding of the Value of Electrotherapy in This Disease and a More Rational, Optimistic Prognosis and Method of Treatment*Read before the Annual Meeting of the American Electro-Therapeutic Association at Atlantic City, September 14-17, 1915.

**Published:** 1916-09

**Authors:** Ludvig A. Brustad

**Affiliations:** San Antonio, Texas


### Infantile Paralysis—A Plea for the Better Understanding of the Value of Electrotherapy in this Disease and a More Rational, Optimistic Prognosis and Method of Treatment.*

BY DR. LUDVIG A. BRUSTAD, SAN ANTONIO, TEXAS.

It is not my purpose to go into the etiology or treatment of acute, anterior poliomyelitis—the epidemics of recent years having called the attention of many abler and more scientific minds to that phase of this dread disease. And whether the *cimex lectularius* is the real agent in the transmission of the infection—although no doubt he has an able ally in the busy *stomoxys calcitrans*—the paralysis remains and the tiny human sufferers are condemned by the majority of our profession to go through life

*Read before the Annual Meeting of the American Electro-Therapeutic Association at Atlantic City, September 14-17, 1915.

more or less crippled and handicapped by the ravages of this justly called "children's plague."

The purpose of this paper is to bring before you as the representative body of advanced therapeutists once more the fact, well recognized by you all, that in the preparalytic stages, a more favorable prognosis may be expected, if we have recourse to electrotherapy. The propriety of convincing every practitioner of medicine throughout the land, that the paralysis can be arrested in the early stages and in the later stages ultimately cured, and the function restored in nearly all cases by the intelligent administration of electricity is self-evident.

As a proof of this we have but to point to the brilliant results obtained in the many so-called hopeless cases sent to the New York Hospital for Deformities, and the work done by Drs. Frauenthal, Manning, Snow and others of this association. To Dr. Snow we owe the introduction of the use of the static wave current in the treatment of this disease—a most important adjunct in my opinion. The attitude of the profession in general towards electrotherapy, and in this disease particularly—is still, to say the least,

"doubtful." It is like the darkie's opinion of the balky mule. "He might go, boss, but he won't and he sholy will kick."

We meet practically every day and we see on all sides of us cripples in all walks and ages of life for whom there should have been a brighter future except for the well meant but mistaken prognosis of some old and trusted family physician. I should say family doctor. From a humanitarian standpoint alone the little ones should have a chance. True, the treatment is prolonged and extends over a period of from one to two years or even longer—but what is this, when compared with a lifetime of invalidism and deformity?

From all reliable statistical reports it is conceded that a little less than sixty per cent of cases have residual paralysis with more or less atrophy of the muscles involved. Spontaneous recovery of function without atrophy averages about sixteen per cent.

I have been unable to find any record of a pathological lesion in the anterior horns of the cord in the cases making a spontaneous recovery. Was not the lesion and subsequent injury gradually repaired by Mother Nature in these cases, and if so, why cannot the same practical result be obtained by instituting early treatment?

The cause of the paralysis is a destruction of the neurons by the invading organism and its toxines and the formation of scar tissue obstructing the impulses, which are entirely lost or finally make a detour through the kindly offices of neighboring neurons to reach their destination; all depending on the area involved. Are the affected neurons entirely destroyed or merely occluded and impeded by the scar tissue? As far as the nerve paths are concerned the obstruction of nerve paths to the different limbs decreases and finally stops contraction of the muscles with consequent atrophy. When we have brought about regeneration of the muscles, and thereby re-established functional action—though Hofer has found normal muscle fiber after the reaction of degeneration was complete, proving beyond a doubt that the muscle fibers are there—nerve impulse is still lacking. If restoration can be accomplished with the electric modalities, then there can be but little if any scar tissue left around our much maligned neuron.

The keynote to all therapy is elimination and stimulation; or, in other words, "stimulate to eliminate." We have stimulation double distilled, concentrated and triturated in electricity. It is the agent *par excellence* for the stimulation of cell tissue, producing the most pronounced effect on metabolism, both anabolically and catabolically. The effect on elimination by the various modalities is well known to you all. After the paralysis has become complete, or rather the obstructing scar tissue has formed, the absorption of the lesion requires a much longer time, which affords a most potent argument for early treatments during the period of infiltration.

I heartily agree with Drs. Snow and Frauenthal that the treatment can be commenced at any time, even before the temperature declines.

The bactericidal effect of the high frequency current in the body may not be as much as claimed; but that current is sure to stimulate metabolic processes generally, and particularly the cell defenders (phagocytes) which will, consequently, be enabled to resist and overcome more readily the invading hosts. To repair the destroyed territory and restore the affected area will take more time.

In biological research it was long ago discovered by Chanoz, Waller and others that in every living cell and tissue there is a demonstrable current of varying potential. May not this be the source of all force? Nerve impulses are but the effect of this force. Are not these impulses known to us as electric energy? The logical remedy for the restoration and repair of diseased nerve function is, therefore, electricity.

We have all of us been asked many times "how does electricity cure?" or, "how does electricity do these things?" And our answers have been manifold according to the intelligence of ourselves and the questioner. My answer is: In the same way that the sunlight makes the plant grow and the flowers bloom, or, to use a familiar parallel, in the same way that the sunlight brings the oak from the acorn. Nothing has been added and nothing has been taken away but certain substances or forces already present have undergone new combinations to which is due the transformation. No doubt a great many of you can answer the question at a greater length and more explicitly than I.

Sunshine is one of our least used but most powerful remedial agents; only it is too cheap. There is no need for me to enumerate the cures effected and diseases treated most successfully by sunlight, except to say that the sun-bath, high power or leucodescent light systematically given, in cases of infantile paralysis will shorten the treatment and improve the condition of your patient.

In giving X-ray treatments I have noticed, as no doubt many of you have, that there is an appreciable static charge of electricity generated or induced in the body of the patient.

I am at present using graduated doses of the X-ray with this object in view, but it is too early for me to report any results. My method of treatment at the present time consists of constant current or sinusoidal applications to motor points of the affected muscle alternating every other day with the static wave current applied to the spine and X-ray once a week.

I have had as the result two complete functional cures within the past year, the treatments averaging from four to eight months. Both cases had already muscle atrophy. The ages of the patients were three and one-half and four years, respectively. The acute attack occurred in one at the age of one year and in the other at eighteen months.

The difficulty seems to be to convince parents that continued treatment over an extended length of time is necessary, so many will be expecting phenomenal results in from one to two months, and failing in that give up, and revert to the idea that the case is hopeless, which idea, I regret to say, is very often fostered and incubated by a great many medical advisers who are consulted by the parents.

My radiologist, Dr. Frederic Johnson, who is a most painstaking and scientific investigator, has been working along the line of endeavoring to measure the ray, a suggestion that may be worth following up.

There is then a vibration of the different body cells set up by the ray in addition to the destructive effect of the X-ray on all living cells—the weaker or diseased ones succumbing first. The effect is, therefore, to dissolve cicatricial tissue or scar formation of lower vitality. We have a powerful agent in the X-ray for the destruction of foreign and diseased cells. Poliomyelitis is an affection of the nerve cells, and it seems logical that the proper use of the X-ray will have an appreciable result in hastening repair in this disease. Does a mild dose of the rap give a stimulus to normal body cells—or does it by further weakening the abnormal or diseased cells hasten their absorption and elimination by the body scavengers? I am inclined to the latter view—but also believe that the current induced in the body of the patient furnishes a stimulus to the system in general.

It is to be hoped that in the coming years, when the large colleges take up the teaching and study of electrotherapeutics, the attitude of the profession will gradually change from a blissful ignorance to a useful wisdom. For the present let us say to our doubting brother—try it once anyhow, but be sure you give it an intelligent trial in justice to yourself and your patient.

---

"Why did that young man look so cross when Mrs. Smith told him that she heard he had such killing ways?"

"She told him that? Great Scott! He's a doctor."—*Baltimore American.*

